# Patient-Reported Outcome Measures in Clinical Practice for Tooth Wear: A Literature Review

**DOI:** 10.3390/jcm14144816

**Published:** 2025-07-08

**Authors:** Inês Argolinha, Sofia Lobo, Ana Vieira, João Botelho, João Rua, José J. Mendes, Vanessa Machado

**Affiliations:** Egas Moniz Center for Interdisciplinary Research (CiiEM), Egas Moniz School of Health and Science, 2829-511 Almada, Portugal; slobo@egasmoniz.edu.pt (S.L.); asvieira@egasmoniz.edu.pt (A.V.); jbotelho@egasmoniz.edu.pt (J.B.); jrua@egasmoniz.edu.pt (J.R.); jmendes@egasmoniz.edu.pt (J.J.M.); vmachado@egasmoniz.edu.pt (V.M.)

**Keywords:** tooth wear, patient-centered outcomes, self-report, oral health-related quality of life

## Abstract

Tooth wear is a growing oral health concern with implications for function, esthetics, and psychological well-being, ultimately affecting oral health-related quality of life (OHRQoL). While clinical indices assess tooth wear severity, they fail to capture patient-reported outcomes (PROs) and patient-reported outcome measures (PROMs). This narrative review aims to identify and synthesize the use of PROs and PROMs used in adults with tooth wear and to map their assessed domains against the Wilson and Cleary model of health outcomes, highlighting gaps and guiding the development of condition-specific instruments. A comprehensive search of the literature was conducted across PubMed, MEDLINE, and Embase. Studies involving PROMs in adults with tooth wear were included. Extracted data encompassed psychometric properties and domains assessed. PROMs such as the OHIP, OES, OIDP, and QMFQ have been frequently used, focusing on functional limitation, esthetic perception, and psychological distress. However, no single instrument comprehensively addresses all relevant domains of the Wilson and Cleary model. Moreover, variation in tools and constructs limits comparability across studies and clinical settings. Existing PROMs capture only partial aspects of the patient experience related to tooth wear. When mapping these instruments to a validated theoretical model, significant gaps become evident, especially in terms of general health perceptions and overall quality of life metrics. To improve the evaluation and management of tooth wear in clinical settings, it is essential to create a condition-specific PROM based on a solid conceptual framework.

## 1. Introduction

Tooth wear is an age-related phenomenon [1] characterized by an irreversible and cumulative surface loss of mineralized tooth structure due to physical or chemo-physical processes unrelated to dental caries [2]. The concept of tooth wear encompasses a multifactorial and complex etiology [3], linked with dental erosion, attrition, and abrasion which can occur either independently or in combination with one another [4].

This wear condition is increasingly acknowledged as a public health concern, affecting 57.1% of European young adults and becoming severe with age [5]. The earliest signs are subtle, involving the loss of dental surface features such as cingula, mamelons, and the smoothing of the facial surface [1]. As the condition progresses, clinical manifestations become more pronounced, potentially leading to dentine hypersensitivity, functional impairment, such as difficulties with chewing due to occlusal alteration and dental tissue loss, and the impairment of esthetics due to shortened clinical crown length. However, patient characteristics play a more significant role in determining the perceived impact of tooth wear than the actual extent of the wear itself [1,6,7]. Consequently, research increasingly acknowledges that the clinical severity of tooth wear does not directly correlate with its perceived impact on aspects of life, including physical, psychological, and social well-being [6,8].

The ability of a patient to recognize the signs and symptoms of tooth wear is crucial, as early detection may prompt individuals to seek timely intervention or treatment. If patients identify symptoms at an early stage, they may access care sooner, potentially improving long-term outcomes. Likewise, when dental professionals understand patients’ perceptions of their oral health, they can enhance communication strategies, promote awareness, and facilitate informed decision-making regarding treatment needs and the consequences of delayed care. In this context, patient-reported outcomes (PROs) refer strictly to the outcomes or domains reported directly by patients, and they play a critical role in capturing the patient’s perspective on their health and its impact (like pain, function, esthetics, or emotional well-being) [9]. These outcomes are best assessed using standardized, validated questionnaires known as patient-reported outcome measures (PROMs) [10,11,12].

PROMs are broadly categorized into generic and condition-specific instruments. Generic PROMs assess overall health aspects, including physical, emotional, and social well-being, across various patient populations [13]. Condition-specific PROMs capture health dimensions relevant to a particular patient group or condition, evaluating symptoms, complications, and treatment-related factors specific to that context. While these instruments may offer enhanced sensitivity, specificity, and responsiveness to changes, they generally have a more narrow focus [13].

The application of PROMs in patients with tooth wear has been attempted, primarily focusing on Oral Health-related Quality of Life (OHRQoL), Orofacial Esthetic Scale (OES) [14], and functional aspects such as Oral Impacts on Daily Performance (OIDP) [15], Dental Impacts on Daily Living (DIDL), and the Quality of Masticatory Function Questionnaire (QMFQ) [16]. Despite their relevance, these instruments encompass both overlapping and distinct domains, reflecting variations in the constructs they assess.

In this sense, a comprehensive synthesis of PROMs used in patients with tooth wear is lacking. To address this gap, we adopted the Wilson and Cleary [17] model of health outcomes, a well-established framework that links biological and clinical variables to symptom status, functional status, general health perceptions, and overall quality of life. This model has demonstrated its usefulness in the realm of health and enables a systematic assessment of whether existing PROMs accurately capture the multifaceted impact of tooth wear from the patient’s viewpoint [18].

We hypothesize that currently available PROMs applied to patients with tooth wear do not fully encompass the outcome domains defined in the Wilson and Cleary model of health outcomes, particularly in areas such as general health perceptions and overall QoL. Therefore, this narrative review aims to identify and synthesize PROMs used in adult patients with tooth wear and to map their assessed domains against the Wilson and Cleary conceptual model of health outcomes. Through this approach, we seek to highlight existing strengths and limitations in current PROMs and to identify essential, underreported domains. We organized the following sections according to each identified instrument.

## 2. Material and Methods

This narrative review employed a structured methodology to enhance transparency and reproducibility.

### 2.1. Literature Search Strategy

A comprehensive search was conducted across three electronic databases: PubMed, MEDLINE, and Embase, covering the period until November 2024. The following keywords and Boolean operators were used in various combinations: “*tooth wear*“, “*dental erosion*”, “*attrition*”, “*abrasion*”, “*patient-reported outcomes*”, “*PROMs*”, “*oral health-related quality of life*”, “*OHRQoL*”, and “*self-report*”. No restrictions were applied in terms of publication date or language.

### 2.2. Eligibility Criteria

To be eligible for inclusion in this review, studies had to fulfill the following criteria: they needed to be original research articles, including feasibility or pilot studies; involve adult participants aged 18 years or older; and report on PROs or PROMs related to tooth wear diagnosis, including erosion, attrition, and/or abrasion.

Studies were excluded if they focused exclusively on pediatric populations; if they were review articles, editorials, case reports, or conference abstracts; or if the full text of the article was not available.

### 2.3. Data Extraction

From each included study, the following data were extracted: study design, sample characteristics, PROM(s) used, domains assessed, psychometric properties (e.g., validity and reliability), clinical indices of tooth wear, and the main findings. Instruments lacking psychometric data or domain-specific application to tooth wear were excluded.

## 3. Results

### 3.1. Study Selection Process

The initial search retrieved 8556 records. After removing duplicates, 7929 titles and abstracts were screened (using Rayyan System Inc., Doha, Qatar; version 2025) by two independent reviewers. Discrepancies were resolved through discussion or by consulting a third reviewer. A total of 120 full-text articles were assessed, resulting in 26 studies meeting the eligibility criteria for inclusion.

From these studies, ten major PROMs were identified as instruments used to evaluate the impact of tooth wear from the patient’s perspective. In the following sections, we describe each PROM in detail.

Additionally, a comprehensive comparative table summarizing the key characteristics of each PROM is provided in the Supplementary Materials (Tables S1 and S2). This visual synthesis supports the narrative by facilitating comparison across tools and highlighting their respective strengths and limitations.

### 3.2. Oral Health Impact Profile (OHIP)

The OHIP is a self-administered tool designed to assess OHRQoL by evaluating how oral health conditions impact an individual’s daily activities, well-being, and overall quality of life (QoL). Although it does not reflect the clinical oral status, it captures the individual’s perception of their oral health and its significance in their daily life [19]. While its original version comprises 49 items (named OHIP-49), its extensive number of items and the time required to complete it limit its feasibility in epidemiological and clinical settings. To enhance practicality, shorter versions such as the OHIP-26 and OHIP-14 were developed to maintain the conceptual integrity of the full version while reducing respondent burden [20,21]. As such, OHIP has been widely used in epidemiological and observational studies to assess the impact of tooth wear on OHRQoL, which are described below according to epidemiology and intervention.

#### 3.2.1. Tooth Wear and OHIP: Epidemiological Perspective

A study by Daly et al. [22] assessed 835 university students (18–30 years, mean age 21.9 ± 0.1), excluding dental students, using OHIP-49 to evaluate the relationship between dental surface loss and QoL. Tooth wear, measured by the tooth wear index (TWI), was highly prevalent among young adults (39% exhibiting mild, 14% moderate, and 47% severe wear, and 77% of participants presented at least one surface with exposed dentine). Despite this, the overall impact on OHRQoL was minimal, with significant differences observed only in the functional limitation domain (*p* = 0.003). Appearance (*p* = 0.01) and discomfort during mastication (*p* = 0.008) were notably affected by the erosion into dentine. These findings suggest that young individuals may be more adaptable to early-stage tooth wear or may not yet experience substantial functional impairments. Moreover, they raise concerns about whether the OHIP-49 is sensitive enough to capture the full impact of tooth wear, underscoring the need for condition-specific assessment tools.

In a large-scale Brazilian population-based study, Goergen et al. [23] investigated the link between 11 oral health conditions with OHRQoL, using OHIP-14, in 1022 adults (626 women and 397 men) aged 35 and older (mean age of 52.5 ± 11.8). The study revealed that the mean score of OHIP was 9.2 ± 9.7 and indicated that xerostomia, halitosis, dentine hypersensitivity, and dental caries were the strongest contributors to reduced OHRQoL. Tooth wear (diagnosed with Basic Erosive Wear Examination [BEWE]), gingivitis, or non-carious cervical lesions showed no significant association with OHRQoL. These findings suggest that some oral health conditions exert a stronger influence on perceived life quality than others, and tooth wear alone may not always lead to substantial impairment.

Similarly, Kanaan et al. [24] examined 570 Belgian dentate adults (median age 52, range: 18–86; 306 women and 264 men) to assess the link between tooth wear, based on BEWE, and OHRQoL using OHIP-14. Tooth wear prevalence was high (75% BEWE ≥ 1), with a higher proportion of males (*p* = 0.02) and individuals over 35 years old (*p* < 0.0001). Nevertheless, the direct impact of tooth wear on the OHRQoL was limited (OR = 1.2; *p* = 0.361) to the patients exhibiting pathological tooth wear; however, the direct impact of tooth wear severity on OHRQoL was limited (OR = 1.2; *p* = 0.361). Instead, tooth sensitivity (OR = 2.9; *p* < 0.001), perception of esthetic changes (OR = 5.9; *p* < 0.001), and barriers to accessing dental care due to cost (OR = 3.6; *p* = 0.002) were stronger predictors of impaired OHRQoL. This underscores the functional and psychosocial effects of tooth wear, even in cases where severity alone may not directly affect OHRQoL.

A nationwide study in the United Kingdom by Li & Bernabé [25], involving 5654 adults (3063 women and 2591 men), revealed that 61.7%, 13.3%, and 1.6% of participants experienced mild, moderate, and severe tooth wear, respectively. They identified a dose–response relationship between tooth wear severity and OHRQoL impairment. Individuals with severe tooth wear had a significantly higher OHIP-14 total score (RR: 1.90; 95% CI: 1.32 to 2.75) compared to those without tooth wear. Furthermore, psychological discomfort (RR = 1.47) and psychological disability (RR = 1.65) were significantly elevated in those with severe wear. For each additional tooth with severe wear, OHIP-14 scores in psychological discomfort and psychological disability domains increased by 1.15 and 1.18 times, respectively. These findings reinforce the idea that esthetic and psychosocial concerns strongly influence the perceived impact of tooth wear on OHRQoL.

Expanding this perspective, in 2012, Papagianni et al. [26] used the Dutch version of OHIP-49 (the OHIP-NL) to compare the OHRQoL of different patient groups, including those with tooth wear (*n* = 51, 21 women and 30 men), temporomandibular disorders (TMD) (*n* = 46, 42 women and 4 men), complete denture wearers (*n* = 43, 16 women and 27 men), and healthy controls (*n* = 58, 41 women and 17 men). Patients with tooth wear had significantly worse OHIP-NL scores than the controls (*p* < 0.001), with their OHRQoL impairment being comparable to that of completely edentulous individuals. However, their scores were still better than those with painful TMD, indicating that while tooth wear negatively affects OHRQoL, it may not be as debilitating as chronic pain conditions.

Mehta et al. [27] assessed tooth wear using BEWE in a multinational study from the United Kingdom, Malta, and Australia. With 319 dentate adults (147 males and 172 females; mean age 42.6 ± 17.1), the BEWE mean score was 6.7 ± 4.4, with 68%, 24.5%, and 7.5% of participants diagnosed with a BEWE score of 8 or less, between 9 and 13, and 14 or over, respectively. Also, the mean OHIP-26 score was 1.84 ± 0.59, and the effect of tooth wear in OHRQoL was 0.028 (*p* = 0.002), demonstrating that greater tooth wear severity was linked to worsening self-reported oral health outcomes. When analyzing the OHIP-26 subdomains, the most affected domains were functional limitation (chewing and speech) (*p* = 0.028), physical discomfort (*p* = 0.027), physical disability (*p* = 0.033), and psychological disability (*p* = 0.027). Interestingly, geographical differences emerged, with Australian participants (who were older on average; 51.4 ± 18.1) reporting lower negative impacts on OHRQoL, possibly reflecting a reduced perception of oral health concerns with aging.

Similarly, Ngoenwiwatkul et al. [28] examined the relationship between oral health status and OHRQoL in 385 elderly individuals (aged 60–93 years, 107 men and 278 women) in Thailand, using OHIP-49. A 63.6% prevalence of tooth attrition was reported, with a mean OHIP-49 score of 54 ± 26. Individuals with more severe tooth wear had significantly worse OHIP-49 scores, indicating poorer perceived oral health (*p* < 0.001). The study highlighted that tooth attrition, tooth sensitivity, gingival swelling, and oral ulcers were major contributors to impaired OHRQoL, reinforcing the functional and discomfort-related burden of tooth wear in older adults.

A unique study conducted by Kumar et al. [29] in India assessed the impact of occupational dental erosion among 400 battery factory workers exposed to sulfuric acid vapors (average age 43.1 ± 8.0). Workers in acidic environments had a considerably higher prevalence of dental erosion according to the TWI diagnosis (39.5% vs. 11.5%) and worse OHIP-14 scores (23.9 ± 0.9 vs. 26.1 ± 9.7, *p* < 0.05). This study highlights the importance of occupational safety measures to mitigate work-related dental erosion and its negative effects on OHRQoL.

Additionally, Warsi et al. [30] examined the impact of gastroesophageal reflux disease (GERD) on OHRQoL in a Pakistani cohort multicentric study (*n* = 187; 78 women and 109 men; age 41–60 years). GERD-related dental erosion significantly worsened OHRQoL, particularly in the psychological discomfort, functional limitation, and physical disability domains. These findings suggest that gastric reflux-induced tooth wear extends beyond functional impairments, also affecting psychosocial well-being.

#### 3.2.2. Tooth Wear and OHIP: The Role of Intervention

A few studies explored the effectiveness of restorative therapy for tooth wear and its long-term impact on OHRQoL. Sterenborg et al. [31] assessed 124 adults with moderate to severe tooth wear (TWI ≥ 2) and found that patients in need of restorative treatment had significantly worse baseline OHIP-NL scores (0.8 ± 0.6) compared to those opting for non-restorative management (0.4 ± 0.3). Van Sambeek et al. [7] further highlighted that psychological discomfort was the most affected OHIP domain, suggesting that esthetic and functional concerns play a major role in self-reported OHRQoL impairments. Finally, Kalaykova et al. [32] observed that patients seeking restorative treatment had high baseline OHIP scores (10.6 ± 4.8) related to chewing and eating difficulties, reinforcing the idea that severe tooth wear affects function beyond esthetics.

A one-year follow-up study by Sterenborg et al. [31] demonstrated that full-mouth rehabilitation with composite restorations significantly improved OHRQoL scores (0.3 ± 0.3, *p* < 0.001), while those in the non-treatment group saw no significant changes (0.4 ± 0.4, *p* > 1.0). The greatest improvements were noted in psychological discomfort, emphasizing the role of esthetic and functional restoration. Building on these findings, Van Sambeek et al. [7] conducted a five-year follow-up, confirming sustained OHRQoL improvements post-treatment, particularly in psychological discomfort. Similarly, Kalaykova et al. [32] found that, although masticatory efficiency did not objectively improve, patients perceived significant functional benefits after treatment (*p* = 0.001).

Another study [33] included 18 men and women (mean age: 41.7 ± 10.4 years) with moderate to severe generalized tooth wear, presenting functional impairments and seeking treatment. OHRQoL was evaluated at baseline and one year post-treatment using the OHIP-NL questionnaire. The mean OHIP score at baseline was 2.0 (±0.6), which significantly decreased to 1.3 (±0.2) after one year (*p* < 0.001), indicating a marked improvement in OHRQoL. The 95% CI for the difference ranged from 0.4 to 1.0, confirming the statistical significance of the observed change.

This single-center randomized controlled trial (RCT) from the Radboud Tooth Wear Project investigated the impact of restorative treatment and the use of an acrylic full-arch removable appliance on OHRQoL and freeway space in patients with moderate to severe tooth wear (TWI ≥ 2). The results showed a significant improvement in OHRQoL after both 1 month (*p* < 0.03) and 1 year (*p* < 0.001), regardless of removable appliance use. While the removable appliance had no significant additional effect on OHRQoL, it was associated with a lower reduction in freeway space after 1 year (*p* = 0.01, 95% CI: 1.09 to −0.15). The study highlights the effectiveness of restorative treatment in improving both functional and subjective outcomes but questions the necessity of pre-treatment vertical dimension of occlusion testing with a removable appliance for optimizing OHRQoL improvements [34].

A German study [35] assessed changes in OHRQoL with the German version of the OHIP-49 following full-mouth rehabilitation for moderate to severe tooth wear, comparing ceramic and CAD/CAM composite restorations. Among 29 patients (mean age: 44.6 ± 28.4 years), significant improvements were observed in appearance (*p* < 0.001), while linguistic limitations showed the least improvement (*p* < 0.001). No significant differences were found between the two materials in appearance (*p* = 0.913), oral function (*p* = 0.349), psychosocial impact (*p* = 0.419), linguistic limitations (*p* = 0.303), or orofacial pain (*p* = 0.347). These results suggest that both materials offer comparable benefits, with esthetics showing the greatest impact on OHRQoL.

Van Sambeek et al., in a systematic review [36], investigated the impact of tooth wear management on OHRQoL in adult patients. The objective was to assess whether restorative or non-restorative treatments for moderate to severe tooth wear led to measurable changes in OHRQoL. The review included six clinical studies, comprising two RCTs and four non-randomized studies (prospective or retrospective), conducted in three countries: the United Kingdom, Germany, and the Netherlands. The findings indicate that restorative treatment (using materials such as composite resins and ceramics) significantly improved OHRQoL. In contrast, counseling and monitoring without restorative intervention did not result in noticeable changes after one year. Additionally, some studies reported a slightly negative esthetic impact in the long term, potentially due to discoloration and wear of restorative materials. However, the studies exhibited methodological limitations, including a high risk of bias in non-randomized studies and the inability to blind participants in RCTs. The main limitation of this review was the heterogeneity of the included studies, particularly in terms of the variation in tooth wear indices used, OHRQoL assessment methods, and follow-up periods.

Overall, evidence indicates that tooth wear substantially impacts OHRQoL, with severity, psychological factors, and functional impairments playing key roles. While some populations adapt to the initial stages of tooth wear, severe cases consistently impair the QoL. Moreover, the effect of tooth wear on OHRQoL may diminish with age since older adults often have lower expectations and functional requirements for their teeth, placing a greater emphasis on general health issues rather than oral conditions. Furthermore, restorative dental procedures show considerable and enduring enhancements in OHRQoL, especially in psychological and functional aspects.

### 3.3. Orofacial Esthetic Scale (OES)

A study conducted in 2009 [37] suggested that when the OHIP-49 is utilized in clinical research, and a detailed assessment of dental appearance is required, incorporating an additional esthetics module is a sensible approach. The OES PROM, created in 2010 [38], was developed to address the need to evaluate patients’ self-perception of esthetics.

Like other questionnaires, the OES has also been translated into different languages, demonstrating the growing interest in this scale [39].

The OES consists of an eight-item questionnaire and aims to evaluate the patient’s self-perceived orofacial esthetics with good reliability and validity in prosthodontic patients and later validated for the general population [40]. These eight items evaluate appearance, including the face, profile, mouth, tooth alignment, tooth shape, tooth color, gums, and overall impression. Each item is rated using an 11-point numeric scale, ranging from 0 (very dissatisfied) to 10 (very satisfied). The first seven items are summed on this scale to create a total score. A score of 0 indicates extreme dissatisfaction across all items, while a score of 70 reflects maximum satisfaction. The final item, which assesses the general impression of esthetics, is evaluated separately but uses the same 0 to 10 scale. In summary, lower scores indicate greater dissatisfaction and a more negative self-perception of orofacial esthetics [14].

In the Netherlands, Wetselaar et al. [14] examined the psychometric properties of the Dutch Oral Evaluation Survey (OES-NL) in a group of dental patients (*n* = 583) who were referred to the clinic due to various dysfunctions or pains. The patients self-reported their experiences with tooth wear and were divided into two groups: those with self-reported tooth wear and those without. Among the 583 patients, 413 were women aged between 18 and 80 (mean age: 50.1 ± 15.1 years) and 170 were men aged between 18 and 78 (mean age: 45.3 years ± 14.7 years). Of these patients, only 343 completed the questionnaire twice; among them, 183 (53.4%) reported experiencing tooth wear. The mean OES summary score for the entire population was 0.79 (range: 0.75–0.83). In the tooth wear group, the mean score increased to 0.82 (range: 0.77–0.86), while the non-tooth wear group had a mean score of 0.75 (range: 0.67–0.81). The overall impression scores further supported these findings, with the tooth wear group showing a mean score of 0.79 (range: 0.73–0.84) compared to 0.76 (range: 0.68–0.82) in the non-tooth wear group. Statistical analysis indicated a significant difference between the tooth wear and non-tooth wear groups for both the OES summary scores and the overall impression scores (*p* < 0.05). Individuals without tooth wear consistently had higher scores than those with tooth wear. Therefore, the authors concluded that the OES-NL is a suitable instrument for assessing esthetics in this population.

In a study [31] assessing the impact of dental esthetics on QoL, the OHIP-NL and OES-NL questionnaires were administered simultaneously to patients with moderate to severe tooth wear (TWI ≥ 2). The study aimed to evaluate whether significant differences in the perception of dental esthetics existed between patients who received only counseling and monitoring and those who underwent restorative treatment. A total of 124 patients (98 men and 26 women, mean age: 40.5 ± 9.3 years) participated. The Counseling and Monitoring Group comprised 46 patients (37 men and 9 women, mean age: 40.8 ± 9.3 years) with a mean maximum TWI score of 2.9 ± 0.5. At baseline, the mean OES summary score was 48 (± 7.0), and the OES overall impression score was 7.1 (± 1.2). After one year, no significant changes were observed in this group’s perception of dental esthetics (*p* = 0.12 for the summary score, *p* = 0.71 for the overall impression score). The Restoration Group comprised 78 patients (61 men and 17 women, mean age: 40.3 ± 8.5 years), with a mean maximum TWI score of 3.3 ± 0.4. At baseline, the mean OES summary score was 38 (± 10), and the OES overall impression score was 5.9 (± 1.5). In contrast to the Counseling and Monitoring Group, patients who received restorative treatment significantly improved their esthetic perception (*p* < 0.001 for both scores). The findings suggest that individuals seeking restorative intervention—primarily due to concerns related to esthetics or pain—experienced a marked enhancement in both their OHRQoL and the perceived appearance of their orofacial region following treatment [31].

A study conducted by Sambeek et al. [7] analyzed the total OES-NL scores in 123 patients (97 men, 26 women; mean age: 37.5 ± 8.8 years) with dental wear who underwent restorative treatment with composite resins. The questionnaires were administered at multiple time points: before treatment (baseline) and at 1 month, 1 year, 3 years, and 5 years post-treatment. At baseline, the mean OES-NL score was 41.8 ± 12.7, which increased significantly to 68.9 ± 7.5 immediately after treatment. Following the restorative intervention, a significant improvement of 11.58 points in OES-NL scores was observed (Confidence intervale [CI]: 7.30–15.35; *p* < 0.001), enhancing patients’ perception of their orofacial esthetics. Over the subsequent years, a statistically significant yet minor decline in OES-NL scores was noted (a decrease of 4.86 points, CI: 8.59–1.56; *p* = 0.005). However, no significant differences in esthetic perception were observed between male and female participants. These findings suggest that even with composite resin restorations, the improvements in perceived orofacial esthetics remain sustained mainly up to five years after treatment completion [7].

Another study also used the OES-NL questionnaire in adult patients with moderate and severe tooth wear who required rehabilitative treatment [33]. In this study, summary scores and overall impression scores were assessed at baseline and one year after treatment in a population of 19 individuals. For the summary score, the mean value increased from 29.7 (±9.9) at baseline to 59.3 ± 5.2 after one year, with a mean difference of 29.6 (±12.9) and a 95% CI of 24.4–35.8 (*p* < 0.001). Regarding the overall impression score, the mean value improved from 4.8 (±1.6) at baseline to 8.5 ± 1.0 after one year, with a mean difference of 3.7 ± 0.5 and a 95% CI of 2.7–4.7 (*p* < 0.001). These findings demonstrate a statistically significant improvement in both measures, indicating a positive impact of the treatment on patients’ perceptions of their condition.

The studies reviewed support the use of the OES-NL questionnaire to assess dental esthetics in patients with tooth wear. Results indicate that restorative treatment improves patients’ perception of their dental appearance, whereas counseling alone does not produce significant changes. These findings reinforce the importance of intervention-based treatments in improving both esthetics and QoL.

### 3.4. Oral Impacts on Daily Performance (OIDP)

The OIDP is a validated tool assessing how oral health problems affect an individual’s everyday activities. It evaluates functional, psychological, and social dimensions by measuring the extent to which oral conditions interfere with essential daily tasks such as eating, speaking, smiling, and social interactions. The performance score is calculated by multiplying the frequency score by the severity score for each daily task, ranging from 0 to 25. A higher OIDP score indicates a more pronounced negative influence on life quality [15].

While this tool is very relevant to evaluating the psychosocial and functional impact of oral health on daily life performance, its application for the tooth wear population has been scarce.

Kalsi et al. [6] explored the association between tooth wear, OIDP, and QoL in a heterogeneous sample of 102 participants (age range 18–70 years, mean age 45.1 ± 13.5; 49 females and 53 males). The subjects had a minimum of 20 teeth and exhibited tooth wear, characterized by at least one surface with exposed dentine. The median (IQR) BEWE, generic OIDP, and CS OIDP scores were 12 (11–14), 6.2 (3.0–15.2; range: 0–63.6), and 4.4 (0–9.1; range: 0–49), respectively. The findings demonstrated that tooth wear significantly affects daily activities, particularly eating, speaking, and social interactions. The magnitude of this impact, however, varied based on individual coping mechanisms, adaptation, and the severity of tooth wear. Minor wear did not result in substantial functional limitations, but more advanced cases increasingly impeded essential daily tasks, underscoring the importance of early detection and continuous monitoring.

Additionally, in a study of patients undergoing bariatric surgery, Marsicano et al. [41] conducted a longitudinal study evaluating oral health conditions before and at 3 and 6 months post-surgery and the impact of oral health on QoL using the OIDP. The findings revealed a high prevalence of dental wear at baseline, based on the Dental Wear Index [42], (81.5%, mean 25.4 ± 9.3), which increased to 87.5% (mean 27.4 ± 12.7) and to 100% (mean 32.7 ± 10.2) 3 and 6 months post-surgery, respectively (*p* = 0.012). Despite this exacerbation of tooth wear, OIDP scores improved significantly over time (baseline: 26.0 ± 43.8; 3 months: 15.5 ± 29.0; and 6 months: 2.5 ± 5.4, *p* = 0.029). This seemingly contradictory result suggests that while oral health conditions deteriorated post-surgery, the overall QoL improved owing to significant general health benefits. The study postulated that patients undergoing bariatric surgery might reprioritize their health concerns, attributing less significance to oral health issues as their systemic health improves. A limitation found in this study was the reduction in the population evaluated over the follow-ups, which went from 54 patients to 24 patients at 3 m and 16 individuals at 6 m.

Overall, studies highlight that tooth wear can impair eating, speaking, and smiling, with psychological factors moderating these effects. Nevertheless, health priorities may shift in certain populations, altering how individuals perceive oral conditions’ impacts over time.

### 3.5. General Health Questionnaire-12 (GHQ-12) and Oral Impact on Daily Performance (OIDP)

The General Health Questionnaire (GHQ) is a self-administered screening tool designed to assess general psychological well-being and distress [43]. Initially, the GHQ consisted of 60 items (GHQ-60), and later a shorter version was created (GHQ-30 and 28) [44]. The 12-Item General Health Questionnaire (GHQ-12) is the most widely used screening tool for common mental disorders, and it also serves as a general measure of psychiatric well-being. A higher GHQ score suggests decreased general psychological well-being. The GHQ evaluates symptoms of anxiety, depression, social dysfunction, and loss of confidence, making it particularly relevant for exploring the psychological impact of chronic dental conditions, including tooth wear.

In the only study found in which this questionnaire was used in patients with tooth wear, Kalsi [6] explored the relationship between generic and condition-specific (CS) QoL, general psychological well-being, and personality in patients with tooth wear. To gather data, researchers used several questionnaires, including the OIDP to assess QoL, the NEO-FFI Personality questionnaire to evaluate personality traits, and the General Health Questionnaire-12 (GHQ) to measure psychological well-being. Tooth wear severity was assessed using the BEWE index (median score for this population was 12). In this study, the median GHQ score was 1 (IQR: 0–5), ranging from 0 to 12, and there was no significant correlation between GHQ scores and age and no notable differences in GHQ scores between men and women (*p* > 0.05). In addition, Spearman’s rank correlation coefficient showed a strong positive relationship between OIDP scores (both generic and condition-specific) and GHQ scores (*p* = 0.000). This means that as the QoL declined—whether in general or due to the specific condition—psychological well-being also decreased. Regression analysis indicated that general psychological well-being, as measured by the GHQ, significantly impacted QoL, even after adjusting for the severity of tooth wear. Specifically, for every 1-unit increase in the GHQ score, the CS OIDP score increased by an average of 1.3 units (95% CI: 0.824–1.827; *p* = 0.000). These results suggest that both psychological well-being and the neuroticism dimension of personality independently influence the QoL, regardless of the severity of tooth wear.

### 3.6. Dental Impacts on Daily Living (DIDL)

The DIDL is a socio-dental instrument that evaluates the impact of oral health conditions on an individual’s daily life and overall satisfaction with their dentition. Unlike other OHRQoL measures, the DIDL incorporates both objective clinical status and subjective patient perceptions, providing a more comprehensive evaluation of how dental conditions affect comfort, function, appearance, pain, and eating ability [45].

Al-Omiri et al. [46] conducted a study assessing the effects of tooth wear [47] on patient satisfaction with their dentition and daily activities using the DIDL. The study included 76 patients with tooth wear (27 women and 49 men, mean age 34.6 ± 9.7) with an average of 25 remaining teeth, and 76 matched controls (mean age 33.0 ± 10.0) with an average of 28 remaining teeth. Results showed that tooth wear patients had significantly lower satisfaction scores across all five dimensions compared to the controls (*p* < 0.001). Notably, 35.5% of patients with tooth wear were dissatisfied with their dentition, compared to only 3.9% of the control group. Dissatisfaction was most pronounced in appearance (76.3%), eating ability (26.3%), and oral comfort (21.1%). Interestingly, no significant correlation between tooth wear severity and patient satisfaction was found (*p* = 0.048), suggesting that factors beyond its clinical severity influence the perceived impact of tooth wear on daily life. Also, age was negatively correlated with satisfaction related to eating (*p* = 0.04), indicating that older individuals may have lower expectations regarding their dentition and may reprioritize oral health concerns over time. These findings highlight that age, sex, and education level can influence perceived satisfaction with oral health, but wear severity does not strongly determine satisfaction levels [44].

### 3.7. Geriatric Oral Health Assessment Index (GOHAI)

The Geriatric Oral Health Assessment Index (GOHAI) is an instrument developed to evaluate self-reported functional issues and psychological effects associated with oral health among elderly individuals. A higher GOHAI score indicates better OHRQoL, while a lower score suggests poorer self-perceived oral health [48].

One study used this instrument to evaluate the relationship between anterior tooth wear and OHRQoL using the GOHAI in a large not-for-profit nursing home population at a facility in the United States. The study by Al-Allaq et al. [49] included 100 participants (63 women and 37 men) who were 55 years and older (mean age: 75.7 ± 11.5 years). Subjects were required to have a minimum of four upper and four lower anterior teeth, either natural or prosthetic, and were deemed cognitively sound. Tooth wear was evaluated using a modified version of the TWI by Donachie and Walls, and GOHAI scores were collected through verbal administration.

The study findings revealed a positive correlation between tooth wear and age, while demonstrating an inverse relationship with QoL. The average GOHAI score among participants was 27.4 ± 7.1, with higher TWI scores associated with increased GOHAI scores (*p* = 0.0003), indicating poorer self-reported oral health. Notably, although tooth wear significantly impacted OHRQoL negatively, age did not show a substantial influence on GOHAI scores (*p* = 0.31). Additionally, women tended to report lower OHRQoL compared to men (*p* = 0.02).

This research pioneered the investigation of the anterior tooth wear’s effect on OHRQoL among nursing home residents using GOHAI, offering a crucial understanding of the connection between functional dental erosion and self-reported oral health in elderly individuals living in care facilities. The investigation achieved a remarkable response rate, with every approached subject consenting to participate, thanks to personalized recruitment methods.

Nevertheless, certain constraints should be acknowledged. The research was limited by its small sample size and single-institution setting, which may restrict the broader applicability of the results. Furthermore, the GOHAI questionnaire was adapted to use a three-point Likert scale (All the time, Sometimes, and Never) instead of the original five-point scale, potentially affecting response patterns. Moreover, the study relied on self-reported data, which can introduce bias, especially among participants with cognitive or communication difficulties.

### 3.8. Quality of Masticatory Function Questionnaire (QMFQ)

The QMFQ is a validated tool for the self-assessment of perceived chewing ability and its effects on OHRQoL [16]. Higher QMFQ scores indicate greater challenges in chewing and a more pronounced negative impact on masticatory function. We can also classify this as low (≤44) or high impact (>44) according to total QMFQ.

One study [50] explored whether tooth wear can modify occlusion and influence chewing efficiency by employing QMFQ to investigate the connection between tooth wear and masticatory performance. This was a cross-sectional study in 197 indigenous Brazilians from the Macuxi tribe in Roraima (Brazil), including adolescents (12–17 years) and adults (18–60 years).

Overall, tooth wear was present in 38.1% of participants using the BEWE scale, with 48.2% reporting an impact on their chewing ability. Tooth wear and self-reported masticatory difficulties were not linked (*p* > 0.05). This suggests that other factors, such as eating habits, occlusal adaptations, and traditional food preparation methods, may have a greater influence on perceived chewing function according to the authors. Age emerged as a strong predictor of tooth wear, with adults being eight times more likely to show tooth wear compared to adolescents (*p* < 0.0001). Dietary habits, including vegetables, oranges, bananas, apples, and lemons, were associated with an increased likelihood of tooth wear. These results underscore the intricate relationship between diet, age, and the progression of tooth wear.

### 3.9. NEO-FFI Personality Questionnaire and OIDP

The NEO-FFI inventory is a validated tool for assessing five core personality traits: neuroticism, extraversion, openness, agreeableness, and conscientiousness. Consisting of 60 items rated on a five-point Likert scale, it provides a concise yet comprehensive evaluation of personality characteristics with strong reliability, validity, and sensitivity [46].

A single study uses the NEO-FFI [6] to analyze the relationship between personality traits in individuals with dental wear. A sample of 102 patients aged 18 to 70 years was assessed using the NEO-FFI questionnaire, along with the OIDP and the GHQ-12. Tooth wear severity was determined using the Basic Erosive Wear Examination (BEWE). Findings showed that higher scores reflect stronger personality traits, with no significant sex differences in neuroticism, extraversion, or conscientiousness (*p* < 0.05). However, women scored significantly higher in openness (*p* = 0.003) and agreeableness (*p* = 0.000). Spearman’s correlation analysis revealed that higher neuroticism was linked to a lower QoL, particularly in eating-related aspects, though these effects were unrelated to tooth wear (*p* < 0.05). However, neuroticism also correlated with a lower QoL in smiling, relaxing, and significant work activities, where the impact was directly associated with tooth wear (*p* < 0.05).

Additionally, individuals with higher openness scores reported lower QoL in emotional well-being and social interactions, though these effects were not directly related to tooth wear (*p* < 0.05). These findings suggest that psychological factors, particularly neuroticism, play a crucial role in the perception of oral health and its impact on daily life, with tooth wear exacerbating these effects in specific domains. Understanding these associations may help develop personalized treatment approaches to improve functional and psychological outcomes in patients with dental wear.

## 4. Discussion

This review adopted the Wilson and Cleary model of health outcomes as a conceptual framework to evaluate how well existing PROMs capture the lived experience of individuals with tooth wear. This model provides a hierarchical structure linking biological and clinical variables to symptom status, functional status, general health perceptions, and overall QoL. By mapping the domains assessed by PROMs to this model, we identified a conceptual imbalance. Most instruments emphasize symptom status (e.g., dentine sensitivity, esthetic dissatisfaction) and functional limitations (e.g., chewing difficulty, speech), while neglecting broader domains such as general health perception and overall well-being.

The selection of a PROM for assessing patients with tooth wear should take into consideration the specific domains, one’s aims to explore the clinical setting, and the characteristics of the patient population. Although the OHIP remains the most commonly used and extensively validated instrument, its generic structure—without items tailored to tooth wear—may reduce its sensitivity to certain aspects, such as the perception of progressive structural loss or early functional compromise. Still, the OHIP-14 offers a pragmatic and time-efficient option for capturing broader aspects of OHRQoL in everyday clinical settings.

When functional limitations are the main concern—especially those interfering with eating, speaking, or social interaction—the OIDP may offer more targeted information. In cases where esthetics and self-image are central to the patient’s concerns, the DIDL may be preferable, as it combines the assessment of function with satisfaction regarding appearance and comfort. For patients undergoing prosthetic or esthetic rehabilitation, the OES can provide valuable insight into perceived facial and dental attractiveness, though its scope is limited to esthetic evaluation.

In older adults, the GOHAI may be more suitable due to its focus on age-related functional and psychosocial aspects. However, its applicability to younger individuals with tooth wear is questionable. When the main objective is to evaluate chewing efficiency from the patient’s perspective, particularly in cases of functional impairment, the QMFQ can be useful. In more severe cases where psychological distress may be present, the GHQ-12—though not oral health-specific—may help identify emotional difficulties associated with tooth wear. Additionally, although the NEO-FFI is not a PROM in the strict sense, it can offer relevant insight into personality traits and coping styles that might influence how patients perceive and adapt to their condition.

While tooth wear is often assessed through its clinical severity and functional consequences, its emotional and psychological impact is just as significant—if not more so—for many patients. Concerns about altered dental appearance, feelings of embarrassment, and diminished self-confidence are commonly reported, and these issues often overshadow the physical symptoms. Notably, the way these experiences are expressed tends to differ between men and women. Men often seek treatment only when function becomes noticeably impaired and may be less likely to acknowledge emotional or esthetic concerns, possibly due to cultural norms that discourage such openness. Women, by contrast, are usually more attuned to even subtle changes in dental esthetics and are more willing to express how these affect their self-esteem and overall well-being. As a result, they may recognize and report the psychosocial burden of tooth wear earlier. These differences underline the importance of using PROMs that are sensitive to sex-related variations, helping clinicians to gain a more accurate understanding of the patient’s lived experience. Moreover, in individuals for whom appearance plays a major personal or professional role, the social and emotional weight of changes in dental esthetics can be even more pronounced. Altogether, these reflections highlight a significant shortcoming, as no single PROM currently encompasses all dimensions of the tooth wear experience. In practice, clinicians must often strike a balance between depth and breadth, sometimes combining multiple instruments to gain a more comprehensive understanding of the patient’s condition.

### Limitations

Our findings should be understood with an awareness of several inherent limitations. First, the heterogeneity of the included studies, including PROM types, sample characteristics, and study designs, limits comparability and generalizability. Second, the mapping of PROM domains to the Wilson and Cleary model was qualitative and interpretative in nature. While conceptually sound, future research should explore quantitative approaches, such as domain overlap analysis or item-level mapping. Finally, many PROMs lacked psychometric validation for tooth wear specifically, like OHIP, which raises concerns about content validity.

Despite these limitations, our approach offers a novel contribution by organizing existing PROMs within a patient-centered health outcome framework. This reinforces the urgent need for the development of a standardized, disease-specific PROM that reflects all key outcome domains relevant to patients with tooth wear.

## 5. Conclusions

Tooth wear exerts a multifaceted impact on patients, extending beyond clinical manifestations to include functional limitations, esthetic concerns, and psychological distress. Notably, women tend to report greater esthetic and emotional concerns despite men exhibiting higher tooth wear scores. This may highlight the complexity underlying the disconnect between clinical severity and subjective patient experience. Restorative treatments using composite resins and ceramics have been effective in enhancing OHRQoL and underscore the value of rehabilitation in moderate or severe cases of tooth wear. Although, concerns about material wear and discoloration raise the need for a long-term follow-up of such treatments coupled with proper patient education on realistic expectations from the treatment. In stark comparison, counseling and monitoring strategies have shown limited benefits, reinforcing the importance of timely intervention.

By mapping these instruments to the Wilson and Cleary model of health outcomes, this review exposes important gaps, particularly in the domains of general health perception and overall quality of life. Psychological well-being and esthetics consistently emerge as core concerns among patients, yet many PROMs underrepresent these aspects or assess them indirectly.

There is a clear need for the development of a standardized, condition-specific PROM for tooth wear. Such an instrument should be grounded in a validated conceptual framework, such as the Wilson and Cleary model, and should incorporate domains that patients consistently report as relevant: symptom severity (e.g., dentine sensitivity), functional status (e.g., mastication, speech), esthetic satisfaction, emotional well-being, and social self-confidence. Additionally, it should be sensitive to sex-related and age differences in perceived impact and validated across diverse patient populations. Once established, such a tool would enhance both clinical decision-making and the evaluation of therapeutic outcomes in a truly patient-centered manner.

## Supplementary Materials

The following supporting information can be downloaded at: https://www.mdpi.com/article/10.3390/jcm14144816/s1, Table S1: Summary of Patient-Reported Outcome Measures (PROMs) Used in Tooth Wear Research; Table S2: Summary of Patient-Reported Outcome Measures (PROMs) in Tooth wear by Questionnaire.
