# Supplementary material for: Patient-Reported Outcome Measures in Clinical Practice for Tooth Wear: A Literature Review
Source: J Clin Med. 2025 Jul 8;14(14):4816. doi: 10.3390/jcm14144816 (PMC12296020; doi:10.3390/jcm14144816)
Supplement: Supplementary file 1 [file jcm-14-04816-s001.zip › jcm-3707300-supplementary.pdf]

Journal of
Clinical Medicine

MDPI

Review

Patient-Reported Outcome Measures in Clinical Practice for Tooth Wear: A Literature Review

Inês Argolinha *, Sofia Lobo, Ana Vieira, João Botelho, João Rua, José J. Mendes and Vanessa Machado

Egas Moniz Center for Interdisciplinary Research (CiiEM), Egas Moniz School of Health and Science, 2829-511 Almada, Portugal; slobo@egasmoniz.edu.pt (S.L.); asvieira@egasmoniz.edu.pt (A.V.); jbotelho@egasmoniz.edu.pt (J.B.); jrua@egasmoniz.edu.pt (J.R.); jmendes@egasmoniz.edu.pt (J.J.M.); vmachado@egasmoniz.edu.pt (V.M.)
* Correspondence: iargolinha@egasmoniz.edu.pt

Table S1. Summary of Patient-Reported Outcome Measures (PROMs) Used in Tooth Wear Research.

PROM	Domains Assessed	Psychometric Properties	Target Population	Clinical Applicability	Strengths	Limitations
**OHIP**	Functional limitation, pain, discomfort, psychological and social impact	High validity, internal consistency, widely validated	General adults; used in tooth wear and other oral conditions	Broad QoL coverage; widely used in research and clinical settings	Extensively validated; versions adapted to setting	Not condition-specific; may miss subtle impacts
**OES**	Facial, dental and gingival aesthetics	Validated; high reliability in general and prosthodontic populations	Adults undergoing aesthetic or prosthetic treatment	Provides insight into daily limitations and satisfaction	Captures change post-treatment; aesthetic-specific	Focus on aesthetics; ignores function/pain
**OIDP**	Daily performances: eating, speaking, hygiene, emotional state	Validated, responsive to functional limitations	General population; applied to wear and systemic health cases	Ideal for prosthetic and aesthetic evaluation in rehabilitative dentistry	Directly measures performance impact; simple scoring	Underused in wear; low sensitivity to mild changes
**GHQ-12**	Psychological well-being (anxiety, depression, self-esteem)	Well-validated screening tool for mental health	Adults with psychological burden; used in wear impact studies	Geriatric-focused; functional and psychological domains	Links mental health to QoL perceptions in wear	Not specific to dentistry; needs contextualization
**DIDL**	Appearance, comfort, pain, eating, speech	Limited validation, early stage use in tooth wear	Adults with functional/aesthetic concerns	Useful in assessing oral condition burden on daily life	Captures satisfaction and emotional domains	Limited data on validity; not widely adopted

**GOHAI**	Function, pain, psychosocial impact (in elderly)	Good internal consistency in elderly; adapted format	Older adults in institutionalized settings	Supplementary for identifying psychological burden in tooth wear	Tailored to elderly needs and comprehension	Three-point scale reduces resolution
**QMFQ**	Perceived masticatory function and chewing difficulties	Validated in adolescents and adults	Indigenous and general populations	Chewing-specific; helpful in dietary and functional assessment	Applicable in epidemiology and public health	No direct link to clinical measures of wear
**NEO-FFI**	Personality traits (neuroticism, openness, etc.)	Validated personality inventory; reliable across populations	Adults with wear and psychological comorbidities	Psychological profiling; supports personalized care	Correlates personality traits with wear perception	Complex interpretation; not a PROM *per se*

Table S2. Summary of Patient-Reported Outcome Measures (PROMs) in Tooth wear by Questionnaire.

Questionnaire	Reported Domain/PRO	Significant Differences vs. Control Group	Source/Study	Score Impact on OHRQoL
**OHIP**	1. Functional limitation<br>2. Physical pain<br>3. Psychological discomfort<br>4. Physical disability<br>5. Psychological disability<br>6. Social disability<br>7. Handicap	1. ✔ chewing, appearance and speech affected<br>2. ✔ pain while chewing, discomfort<br>3. ✔ mild to moderate psychological distress<br>4. ✔ Difficulty performing oral-function-related tasks<br>5. ✔ loss of confidence, emotional impact<br>6 and 7. Not directly reported as significant	1 and 2<br>Daly et al.; Mehta et al.<br>3. Li & Bernabé; Sterenborg et al.; Van Sambeek et al.<br>4 Mehta et al.<br>5. Li & Bernabé; Van Sambeek et al.<br>6. Not highlighted in studies	Higher score = Worse OHRQoL
**OES**	1. Appearance<br>2. Comfort<br>3. Pain<br>4. Eating<br>5. Function	✔ Significant differences in perceived aesthetics before and after treatment	Wetselaar et al.; Van Sambeek et al.	Lower score = Worse OHRQoL
**OIDP**	Orofacial aesthetics	✔ Differences in daily function like eating and speaking based on wear severity	Kalsi et al.; Marsicano et al.	Higher score = Worse OHRQoL
**GHQ-12**	General mental well-being	✔ GHQ scores correlated with lower QoL regardless of wear severity	Kalsi et al.	Higher score = Worse OHRQoL

**DIDL**	Comfort, Function, Appearance, Pain, Eating ability	✔ Lower satisfaction in all domains; no correlation with severity, but influenced by gender, age, education	Al-Omiri et al.	Lower score = Worse OHRQoL
**GOHAI**	1. Anxiety<br>2. Depression<br>3. Social disfunction	✔ Significant association between anterior wear and lower GOHAI<br>✔ Differences between females and males	Al-Allaq et al.	Lower score = Worse OHRQoL
**QMFQ**	Masticatory function	No statistically significant association with tooth wear	Machado et al.	Lower score = Worse OHRQoL
**NEO-FFI**	Personality traits: Neuroticism, Extraversion, Openness, Agreeableness, Conscientiousness	✔ Correlation between high neuroticism and worse perception of tooth wear impact	Al-Omiri et al.	Higher neuroticism score = Worse perceived OHRQoL

Abbreviations:

DIDL – Dental Impacts on Daily Living; **GHQ-12** – General Health Questionnaire – 12 Items; **GOHAI** – Geriatric Oral Health Assessment Index; **NEO-FFI** – NEO Five-Factor Inventory; **OIDP** – Oral Impacts on Daily Performance; **OES** – Orofacial Esthetic Scale; **OHIP** – Oral Health Impact Profile; **QMFQ** – Quality of Masticatory Function Questionnaire.
